# Multiresponsive Behavior of Functional Poly(*p*-phenylene vinylene)s in Water

**DOI:** 10.3390/polym8100365

**Published:** 2016-10-18

**Authors:** Kanykei Ryskulova, Anupama Rao Gulur Srinivas, Thomas Kerr-Phillips, Hui Peng, David Barker, Jadranka Travas-Sejdic, Richard Hoogenboom

**Affiliations:** 1 Supramolecular Chemistry Group, Department of Organic and Macromolecular Chemistry, Faculty of Science, Ghent University, Krijgslaan 281 S4, Ghent B-9000, Belgium; kanikey@gmail.com; 2 Polymer Electronics Research Center, School of Chemical Sciences, The University of Auckland, Private Bag 92019, Auckland, New Zealand; gsanu85@gmail.com (A.R.G.S.); tker016@aucklanduni.ac.nz (T.K.-P.); d.barker@auckland.ac.nz (D.B.); j.travas-sejdic@auckland.ac.nz (J.T.-S.); 3 MacDiarmid Institute for Advanced Materials and Nanotechnology, Victoria University of Wellington, P.O. Box 600, Wellington, New Zealand; 4 Key Laboratory of Polarized Materials and Devices, Ministry of Education, East China Normal University, Shanghai 200062, China; h.peng@auckland.ac.nz; 5 Collaborative Innovation Center of Extreme Optics, Shanxi University, Taiyuan 030006, Shanxi, China

**Keywords:** polymeric sensor, water-soluble conjugated polymer, fluorescent sensor, reversible calcium binding, thermoresponsive polymer, upper critical solution temperature

## Abstract

The multiresponsive behavior of functionalized water-soluble conjugated polymers (CPs) is presented with potential applications for sensors. In this study, we investigated the aqueous solubility behavior of water-soluble CPs with high photoluminescence and with a particular focus on their pH and temperature responsiveness. For this purpose, two poly(phenylene vinylene)s (PPVs)—namely 2,5-substituted PPVs bearing both carboxylic acid and methoxyoligoethylene glycol units—were investigated, with different amount of carboxylic acid units. Changes in the pH and temperature of polymer solutions led to a response in the fluorescence intensity in a pH range from 3 to 10 and for temperatures ranging from 10 to 85 °C. Additionally, it is demonstrated that the polymer with the largest number of carboxylic acid groups displays upper critical solution temperature (UCST)-like thermoresponsive behavior in the presence of a divalent ion like Ca^2+^. The sensing capability of these water-soluble PPVs could be utilized to design smart materials with multiresponsive behavior in biomedicine and soft materials.

## 1. Introduction

Polymers that respond to external stimuli such as pH, temperature, ionic strength, light, and concentration are extensively studied for a broad range of applications, including biomedical applications like drug delivery [1,2], cell imaging [3,4,5], as well as biological [6] and optical sensing [7,8,9]. Polymer-based systems with responsive properties can straightforwardly be designed thanks to recent advances in polymer synthesis as well as better understanding of physicochemical properties at the molecular level [10,11,12]. Such responsive functions arise from the chemical or topological structure of the polymer and lead to the design of stimuli-responsive smart materials with tailored functions. In contrast to their small molecular analogs, macromolecular or polymeric sensors exhibit numerous advantageous features, such as adjustable water solubility, higher detection sensitivity, straightforward integration into sensing devices, better stability, biocompatibility, and sharpened responses [13].

The important role of temperature and pH in many biological processes has led to increased interest in constructing smart materials that respond to these parameters. To develop polymeric sensors for temperature and/or pH, normally two routes can be followed. Either a water-soluble polymer is modified with a temperature- or pH-sensitive dye [3,4,7,14], or a temperature- or pH-responsive polymer is modified with a solvatochromic dye [15,16,17,18] to translate the polymer phase transition into a fluorescent or absorbance output signal. Whereas a variety of polymeric sensors are reported in literature either for pH [19,20,21] or temperature[22,23], only a few dual-responsive polymers for both pH and temperature exist [3,7,24]. A recent emerging strategy to design such dual-responsive system incorporates a pH-responsive solvatochromic dye, such as Disperse Red 1, into a thermoresponsive polymer [3,25].

Conjugated polymers (CPs) are widely applied for sensing applications. CPs provide a platform for prominent detection of chemical and biological entities due to a highly delocalized electronic backbone structure and optical signal amplification that are sensitive to changes in the structure induced by minor quantities of analytes [26,27,28,29]. For biomedical applications and aqueous sensing, water-soluble CPs are required, which can be achieved by introducing ionic or polar functional groups to the side chains, such as sulfonate, phosphate, carboxylate, quaternary ammonium, and ethylene glycol units [8,13]. Such water-soluble CPs have been utilized to develop biosensors for detection of DNA strands [30,31,32,33,34] and proteins [35,36] and for cellular imaging [3,37]. Aggregation induced by the analyte, which results in a change of the spectral signal (absorbance or fluorescence), is the most common method for analyte sensing with CPs [38,39], although aggregation can also lead to fluorescence quenching or signal enhancement [40].

Carboxylate-functionalized conjugated polymers developed for ion sensing demonstrated cation-induced aggregation with divalent cations [38], where quenching is enhanced with cation concentration increase [41]. Fluorescent carboxylated polyelectrolytes, water-soluble poly(*p*-phenylene ethylene) [42] and poly(thiophene) [43], are credited for their high sensitivity in biosensing applications. Sensing by these polymers of viologen and protein calmodulin, respectively, is based upon Ca^2+^ binding, which gives a shift in absorption or emission spectra based on aggregation of polymer or change in conformation.

**Scheme 1:**
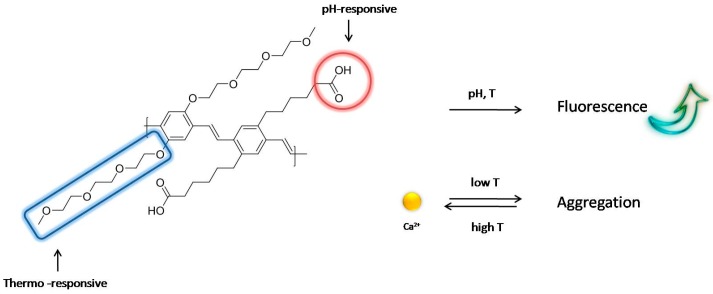
General polymer structure with stimuli-responsive functional units, its response to pH, temperature, and upper critical solution temperature (UCST)-like behavior when complexed with Ca^2+^.

In our recent report [13], we described the synthesis of water-soluble poly(phenylene vinylene)s (PPVs) having both carboxylic acid and methoxyoligoethylene glycol pendant groups (Scheme 1). In the current work, we focused our attention to the multiresponsive sensing behavior of these materials, as the carboxylic acid moiety should bring pH responsiveness while the oligoethylene glycol side chains may induce thermoresponsive behavior. Furthermore, the conjugated polymer backbone may enable a direct visual output signal upon a change in solubility and/or aggregation state of the polymer. Thermoresponsive oligoethylene glycol-modified polymers are gaining increased attention as alternatives for the well-known poly(*N*-isopropylacrylamide) (PNIPAAM) [44,45,46]. Such oligoethylene glycol-modified polymers often exhibit lower critical solution temperature (LCST) behavior in water, meaning that they are water-soluble at lower temperatures and undergo entropy-driven phase separation at a critical temperature. Opposite thermoresponsive behavior, so-called upper critical solution temperature (UCST), whereby the polymer is insoluble at lower temperatures and solubilizes upon heating, is less common in water and requires strong interpolymer interactions [47,48]. UCST behavior can also be induced by combining charged polymers with oppositely charged species, such as metal ions, metal ligand complexes, or organic compounds. A prime example is the UCST behavior of alginate in presence of calcium(II) ions [49,50,51,52,53,54]. Inspired by these systems, we additionally explored whether the acid-functionalized PPVs under study exhibit UCST behavior in presence of calcium(II) analogs (Scheme 1).

## 2. Experimental

### 2.1. Materials

All chemicals were commercially available and used as received unless otherwise stated. Milli-Q water was obtained from a Sartorius Arium 611(Brussels, Belgium) with a Sartopore 2 150 (0.45 + 0.2 mm pore size) cartridge filter (resistivity less than 18.2 MU cm). NaOH and HCl are from Acros Organics (Geel, Belgium) and were diluted with Milli-Q water. Functionalized PPVs were synthesized as previously reported by us [13].

### 2.2. Instrumentation/Methods

The pH was recorded with a Mettler Toledo FE20 FiveEasy Benchtop pH meter (Brussels, Belgium). All spectroscopic measurements were carried out in 1 cm quartz cuvettes. The fluorescence emission spectra were measured on a Cary Eclipse fluorescence spectrometer (Santa Clara, CA, USA) with Peltier temperature control under stirring. The excitation wavelength was set at 392 nm with photomultiplier tube voltage at 600 V. The split width of the excitation and emission were both 5 nm. The fluorescence spectra were recorded with a recording emission range of 400–750 nm. The thermal measurements were taken between 5 and 85 °C with heating/cooling rate of 1 °C/min. Optical absorption (UV–vis) spectra were measured using a Varian Cary 300 Bio UV–visible spectrometer (Santa Clara, CA, USA) equipped with a Cary Peltier temperature control while stirring. Samples were measured in quartz cuvettes with a pathlength of 1.0 cm in the wavelength range of 200–600 nm. The concentration of each sample was 1.0 mg/mL in Milli-Q water.

^1^H-NMR spectra were recorded on a Bruker Avance 400 or 300 MHz spectrometer (Billerica, MA, USA) at room temperature in deuterated solvents. Chemical shifts (δ) are given in ppm relative to TMS.

Size-exclusion chromatography (SEC) was performed on an Agilent 1260-series HPLC system (Santa Clara, CA, USA) equipped with a 1260 online degasser, a 1260 ISO-pump, a 1260 automatic liquid sampler, a thermostatted column compartment, a 1260 diode array detector (DAD) and a 1260 refractive index detector (RID). Analyses were performed on a PSS Gram30 column in series with a PSS Gram1000 column at 50 °C. DMAc containing 50 mM of LiCl was used as eluent at a flow rate of 0.6 mL/min. The SEC traces were analyzed using the Agilent Chemstation software with the GPC add-on. Size exclusion chromatography was used to evaluate the number average molecular weight (*M*_n_) and dispersity (*Ð*) against PMMA standards.

Turbidity measurements were performed on a Crystal16 from Technobis Crystallization Systems (Alkmaar, The Netherlands) at a wavelength of 600 nm. The samples were fully dissolved at pH = 10 (0.5 mg/mL), after which the samples were placed in the instrument and cooled to 10 °C. The transmittance was measured during at least two controlled cooling/heating cycles with a cooling/heating rate of 1 °C·min^−1^ while stirring in PS cuvettes controlled by block temperature probe.

### 2.3. Synthesis and Characterization

#### 2.3.1. Synthesis of 1,4-bis(2-(2-(2-Methoxyethoxy)ethoxy)ethoxy)-2,5-divinylbenzene

To a solution of 1,4-bis(2-(2-(2-methoxyethoxy)ethoxy)ethoxy)-2,5-diiodobenzene (1 g, 1.5 mmol) and vinyltributyltin (1 mL, 3.7 mmol) in DMF (6 mL), triphenylphosphine palladium(0) was added (0.08 g, 0.07 mmol) and the reaction vessel was immediately sealed and degassed via freeze-pump-thaw (ca. 5 cycles). The mixture was then heated to 100 °C and stirred for 6 h. The solution was then allowed to cool before diluting with DCM (ca. 50 mL) and filtering into cold water. The organic layer was then washed three times with water, once with brine solution, dried (Na_2_SO_4_), and reduced via vacuo. This was then purified by passing through a hexane column to yield the title compound, in approximately 40% yield, as a dark red oil.

δH (300 MHz; CDCl_3_; Me_4_Si): 7.25 (2H, s, ArH), 4.18 (4H, t, *J* = 6 Hz, CH_2_), 3.82 (4H, t, *J* = 6 Hz, CH_2_), 3.80–3.75 (4H, m, CH_2_), 3.74–3.68 (8H, m, CH_2_), 3.66–3.49 (4H, m, CH_2_), 3.38 (6H, s, OCH_3_).

IR: νmax(neat)/cm^−1^; 2875 (CH, aromatic), 1352 (C–O, ether), 1242 (C–O, ether), 1176 (C–O, ether), 1096 (C–O, ether) 395 (C–I). *m*/*z* (CI+) 677 (MH+, 100%). High Resolution (CI+): found (MH+): 677.0100 C_22_H_31_O_8_I_2_ requires 677.0103. The 1H NMR data was in agreement with literature values [13].

#### 2.3.2. Synthesis of PMEE-PDTriG (Figure 1)

A solution of tri-*n*-butylamine (0.60 mL, 1.86 mmol), palladium acetate (15.00 mg, 0.02 mmol), tri-*o*-tolylphosphine (80.00 mg, 0.25 mmol), 1,4-bis(2-(2-(2-methoxyethoxy)ethoxy)ethoxy)-2,5-divinylbenzene (0.2 g, 0.44 mmol) and 1,4-diiodo-2,5-dimethoxybenzene (0.36 mmol), 6,6′-((2,5-diiodo-1,4-phenylene)bis(oxy))dihexanoic acid (0.055 g, 0.09 mmol) in DMF (4 mL) under an atmosphere of nitrogen, was degassed using freeze-thaw cycles (×5), before heating at 90 °C for 24 h. The mixture was then filtered and dissolved in basic water, this was then dialyzed against deionized water for 2 days with a 6–8 kD MWCO cellulose membrane. Water was removed in vacuum to give the title compound PMEE-PDTriG, in approximately 50% yield, as a red gel-like solid.

δH (400 MHz; CDCl_3_; Me_4_Si): 7.96 (2H, s, ArH), 7.54–7.51 (2H, m, ArH), 7.29–7.20 (4H, m, ArH), 6.92–6.85 (8H, m, CH=CH), 4.24 (8H, m, OCH_2_), 3.92 (6H, bs, OCH_3_), 3.69 (6H, bs, OCH_3_), 3.58–3.19 (m, OCH_2_, OCH_3_), 2.29 (4H, m, CH_2_), 1.72–1.50 (12H, m, CH_2_).

### 2.4. Sample Preparation

Samples for fluorescent measurements first were dissolved in Milli-Q water at pH = 13 and adjusted to lower pH values by dilute HCl. All samples were prepared at 0.01 mg/mL. For UV–vis measurements, samples were prepared at pH = 10 (0.5 mg/mL) in Milli-Q water (Milli-Q, resistivity ≤18.2 MΩ·cm).

## 3. Results and Discussion

### 3.1. Synthesis and Characterization

**Figure 2:**
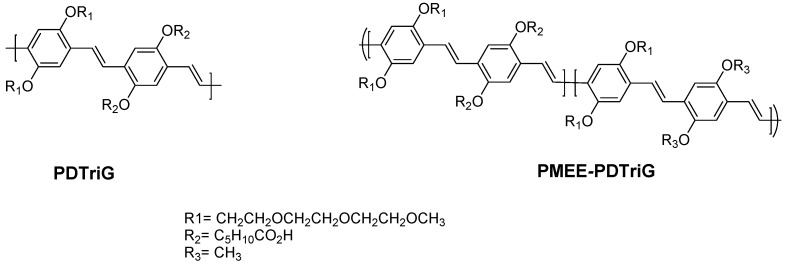
Structures of the polymers: PDTriG and PMEE-PDTriG.

PPVs with functional carboxylic acid and oligoethylene glycol units were previously reported to show high solubility and high luminescence in aqueous and organic media [13]. Here, specifically 2,5-substituted PPV polymers decorated with methoxytriethylene glycol units and carboxylic acid groups (denoted as PDTriG) and its more hydrophobic copolymer with methoxytrietylene glycol and methoxy side groups (denoted as PMEE-PDTriG) were investigated to evaluate their responsive behavior (Figure 2). PDTriG was synthesized as previously reported [13] whereas the monomer and polymer of PMEE-PDTriG were synthesized using a slightly modified literature procedure [13]. These polymers were characterized by means of NMR and SEC. Size exclusion chromatography (SEC) with DMA as a solvent and PMMA as calibration standard was used to obtain the average molecular mass of PDTriG (*M*_n_ = 197,000; *Ð*= 1.34) and PMEE-PDTriG (*M*_n_ = 108,200; *Ð* = 1.04).

### 3.2. Responsive Behavior

#### 3.2.1. pH-Responsive Behavior

**Figure 3:**
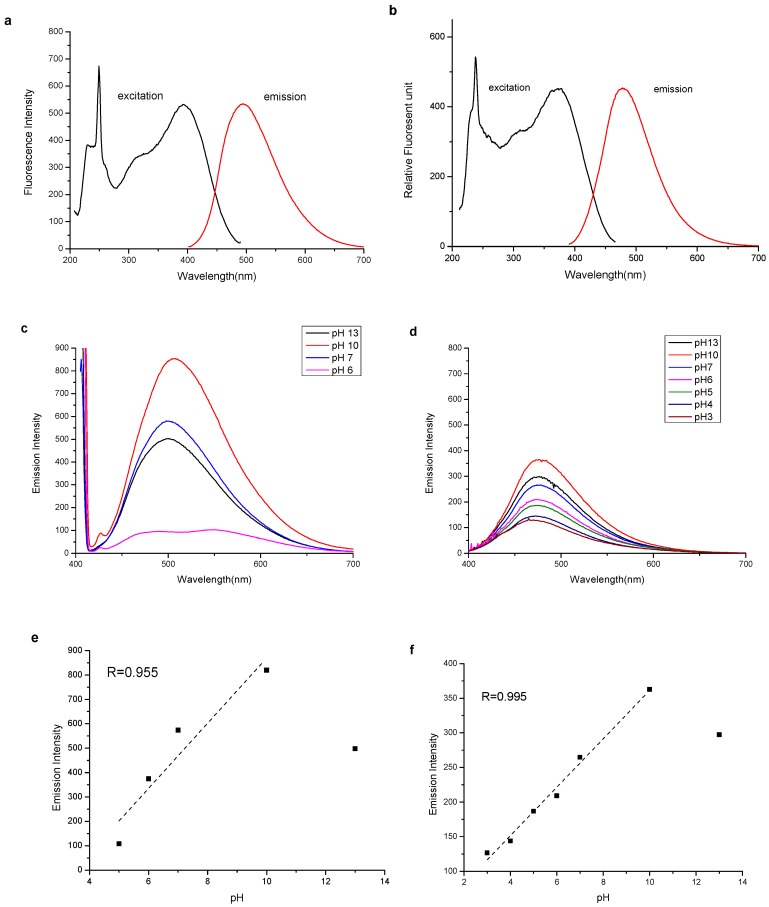
Emission and excitation spectra of (**a**) PDTriG; (**b**) PMEE-PDTriG, measured at pH = 13, 0.01 M. Fluorescence spectra of (**c**) PDTriG; (**d**) PMEE-PDTriG at different pH values at 20 °C, 0.01 mg/mL. Linear fit of fluorescence intensity of (**e**) PDTriG (**f**) PMEE-PDTriG as a function of pH at emission λ_max_.

It is well known that the optical properties of CPs are governed by the polarity of the polymer, conformational changes in the backbone, the polarity of the solvent, intramolecular dynamics, and interchain interactions [55,56]. A change in the conformation of the polymer chain is dictated by different variables, among which solvent and temperature are prominent. Accordingly, any external factor effecting backbone configuration influences the photophysical features of CPs. It was shown previously that the emission spectra of functional PPVs were highly dependent on acidity of the media [13]. Photophysical properties of both PDTriG and PMEE-PDTriG were studied in aqueous media as function of pH by fluorescence spectroscopy. It was observed that the polymers did not readily dissolve in water and in buffer solution, unless the pH was increased to 13. Therefore, the samples were first fully solubilized at pH = 13 followed by addition of dilute hydrochloric acid solution to adjust the pH to the desired pH values. When following this procedure the polymers remained in aqueous solution and no macroscopic precipitate was observed. As shown in the fluorescence spectra in Figure 3a, PDTriG has a maximum emission at 498 nm and an excitation maximum at 392 nm, as measured at pH = 13, 0.01 mg/mL. PMEE-PDTriG was found to have an emission λ_max_ of 478 nm and an excitation λ_max_ of 378 nm when measured at the same concentration and pH (Figure 3b) indicating both polymers have similar optical properties. Thus, all the emission measurements were carried out at an excitation maximum wavelength λ_max_ = 392 nm for PDTriG and λ_max_ = 378 nm for PMEE-PDTriG. The fluorescence intensity of both polymers was found to be dependent on the pH of the solution, as expected based on the presence of the carboxylic acid groups. While at pH below 7 quenching of the fluorescence was observed, strong emission was found at higher pH (pH > 7). This change in emission intensity can be attributed to the enhanced solubility of the polymer at higher pH values due to deprotonation of the carboxylic acid groups, thereby suppressing aggregation of the polymer and quenching of fluorescence [57]. Fluorescence intensity increased for both of the polymers as pH values increased, without shifting of emission maxima peaks (Figure 3c,d). A slight blue shift is observed for PDTriG with decreasing acidity, at pH 6 and below, due to aggregation of amphiphilic CPs as they become protonated [56]. It was, however, observed that the fluorescence intensity of PDTriG decreased in time at pH 13 and at pH 6 and lower. In contrast, PMEE-PDTriG was found to be more stable and only showed a decrease in fluorescence intensity at pH 13 and below pH 3. At lower pH values, protonation of the carboxylic acid groups is presumed to cause insolubility of the polymers; this was proved by complete loss of emission intensity, which was not restored even after adjusting pH value back to 10. Especially, the higher stability at lower pH values for PMEE-PDTriG indicates that the presence of protonated carboxylic acid groups induces degradation and/or insolubility, which is more pronounced for PDTriG as it has more carboxylic acid groups. Note that for sample preparation, the polymers were only dissolved in pH 13 for a short time to avoid degradation of the materials, presumably resulting from the high concentration of hydroxyl anions facilitating degradation of the polymer backbone.

When plotting the fluorescence intensity of PDTriG versus pH, it is clear that no good correlation can be drawn, possibly due to the low stability of the polymers. However, PMEE-PDTriG revealed a linear decrease of fluorescence intensity with decreasing pH in between pH 3 and pH 10, making it suitable as fluorescent pH sensor. This rather broad pH range also facilitates employment in a biological environment as it covers the physiological pH range.

#### 3.2.2. Thermoresponsive Behavior

**Figure 4:**
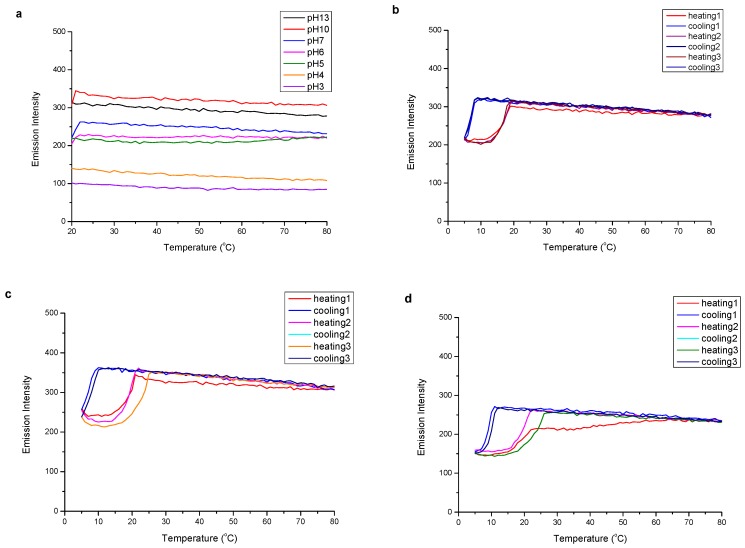
Fluorescence emission as a function of temperature at different pH values (**a**) PMEE-PDTriG. Thermal stability with heating and cooling cycles of PMEE-PDTriG at (**b**) pH = 13; (**c**) pH = 10; (**d**) pH = 7.

As mentioned above, the conformation of the π-conjugated backbone has substantial impact on the photophysical properties of CPs [8]. Therefore, thermoresponsive behavior of the studied PPVs would also induce a change in solubility that also strongly influences the backbone conformation, directly leading to fluorescent output signal. Possible temperature effects on the photoluminescent properties of the more stable PMEE-PDTriG were studied by investigating the fluorescence intensity as function of temperatures, where it was anticipated that these polymers may exhibit LCST behavior based on the methoxyoligoethylene glycol side chains. Fluorescence intensity of the PMEE-PDTriG was measured at different pH values upon heating from 20 to 80 °C, revealing a minor decrease in fluorescence intensity with increasing temperature due to increased chain mobility leading to a decrease in conjugation length (Figure 4). The relative order in fluorescence intensity at different pH values is, however, retained at different temperatures. Nonetheless, this minor temperature influence will make it necessary to recalibrate the pH-sensing response at different temperatures.

A more critical look at Figure 4 reveals some minor disturbances in fluorescence intensity at lower temperature, especially at pH 7. Intrigued by this effect and to probe the stability of the polymer, the fluorescence intensity was recorded during multiple heating–cooling cycles at pH 7, 10, and 13 in between 10 and 80 °C (Figure 4b–d). First of all, these experiments revealed that PMEE-PDTriG is stable during multiple cycles; only the first heating shows a slightly different behavior at pH 7 and 10, most likely related to solubilization since this effect is no longer seen in the second and third heating runs. Secondly, it is evident that PMEE-PDTriG reveals distinct thermoresponsive behavior around 10–20 °C. This abrupt increase in fluorescence emission intensity upon heating is most likely due to LCST-like behavior of the oligoethylene glycol side chains, as such LCST behavior is an abrupt cooperative dehydration effect. More specifically, the observed increase in fluorescence emission intensity may be attributed to (partial) dehydration of the oligoethylene glycol side chains, leading them to collapse. No macroscopic phase separation is observed, as the PMEE-PDTriG remains stabilized and solubilized by the charged carboxylate groups. Interestingly, the reverse process of rehydration of the oligoethylene glycol side chains only occurs around 10 °C, representing some hysteresis in the responsive behavior. This observation may be related to the presence of the hydrophobic alkyl chains on the carboxylic acid side chains facilitating the formation of hydrophobic domains with the dehydrated oligoethylene glycol chains, thereby making it more difficult to rehydrate these oligoethylene glycol chains during cooling.

**Figure 5:**
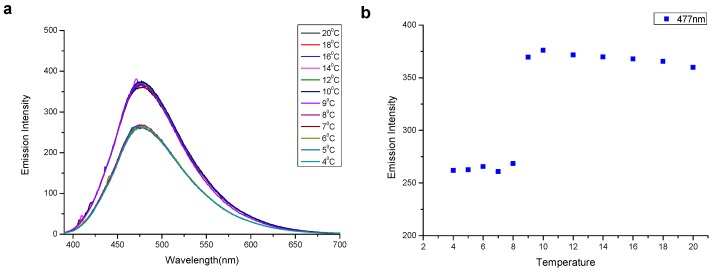
(**a**) Fluorescence emission spectra of PMEE-PDTriG at low temperatures, from 4 to 20 °C; (**b**) emission intensity at emission maxima, λ_max_ versus temperature plot.

To gain further insights into the effect of temperature and, especially, dehydration of the oligoethylene glycol side chains on the polymer chain conformation, full emission spectra were recorded at different temperatures (Figure 5). Changing the temperature, however, showed no shift in emission wavelength maxima but only resulted in a decrease in fluorescence intensity. The low emission intensity at lower temperatures showed a pronounced increase at 9 °C, giving a jump of emission intensity as depicted in Figure 5b. Therefore, it may be speculated that the collapse of the oligoethylene glycol side chains leads to an increase in chain rigidity, thereby increasing the conjugation length that results in enhanced emission intensity. It is noteworthy to mention that the more hydrophilic PDTriG did not show such thermoresponsive behavior.

The observed thermoresponsive emission behavior of PMEE-PDTriG may be utilized for aqueous temperature sensing, whereby the hysteresis gap could lead to a thermal memory of the solution temperature [58,59].

#### 3.2.3. UCST-Like Behavior with Calcium(II) Ions

**Scheme 2:**
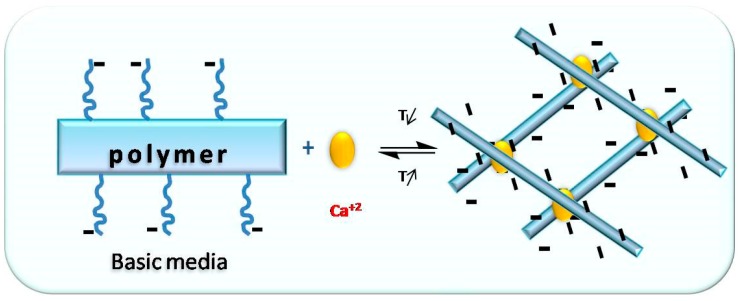
Schematic representation of the reversible binding of PDTriG with CaCl_2_ in basic media leading to UCST behavior.

Besides studying the potential of these functional PPVs as pH and temperature sensors, we were interested to exploit the presence of carboxylate groups for inducing UCST-like thermoresponsive solubility behavior. Therefore, PDTriG was employed as it has a higher load of carboxylic acid groups. The polymer was solubilized in basic solution so that the carboxylic acid groups were present as carboxylate groups, which are known to bind with calcium(II) ions in a 2:1 fashion [60,61,62]. Upon addition of half an equivalent of calcium(II) ions compared to the carboxylate groups, all polymer precipitated out of solution, ascribed to interpolymer crosslinking by calcium(II)–carboxylate coordination as schematically depicted in Scheme 2. By heating up the polymer–calcium(II) precipitate, the supramolecular association is weakened leading to dissolution of the polymer and the calcium(II) ions, thereby providing reversible binding of calcium(II) as function of temperature.

**Figure 6:**
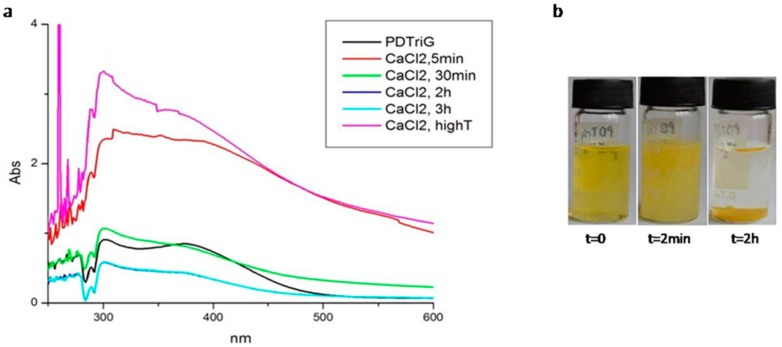
(**a**) UV–vis spectra of PDTriG (pH = 10, 0.5 mg/mL) on addition of CaCl_2_ (1:1 equivalents) in basic media at 25 °C after 5 min (red), after 30 min (green), after 2 h (blue), after 3 h (light blue), and at 60 °C (purple); (**b**) visual observation of complexation within time demonstrating the phase separation of PDTriG in presence of calcium(II) ions.

**Figure 7:**
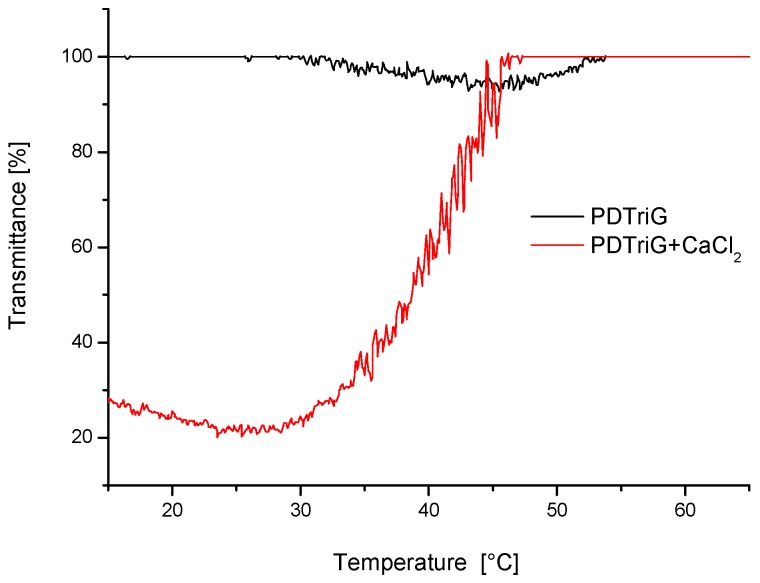
Transmittance as a function of temperature for PDTriG (black) and PDTriG with CaCl_2_ (red) at pH = 10 (0.5 mg/mL) measured at a wavelength of 600 nm.

The complexation and precipitation of the polymer with calcium(II) ions is also shown in Figure 6, indicated by the loss of UV-absorbance as well as by the photographs of the vial. As the precipitated PDTriG–calcium complex is held together by noncovalent supramolecular interactions, we explored whether heating would lead to sufficient lowering of the interaction strength to bring the polymer back in solution. Indeed, heating the sample vial led to regaining of the yellow solution as the precipitate redissolved. Subsequent cooling led to precipitation demonstrating the reversibility of this UCST-like thermoresponsive behavior of PDTriG. Temperature response of the PDTriG and PDTriG–calcium complex is further shown by UV–vis turbidimetry. As depicted in Figure 7, heating the samples from 10 to 70 °C gives no change in the transmittance of the polymer solution, whereas the polymer–calcium complex goes through UCST-like phase transition.

## 4. Conclusions

Functional groups on PPV not only give high solubility in water but also provide new properties for sensing regime of the polymers. In this study, water-soluble PPVs with carboxylic acid groups and methoxyoligoethylene units as pendant side chains are reported to have high luminescence in aqueous media with multiresponsive behavior. Higher solubility is attained for the polymers with increased pH values as more carboxylic acid groups are deprotonated, thereby inhibiting aggregation of the polymers and enhancing fluorescence intensity. This phenomenon is observed for both of the polymers, providing pH response behavior of PDTriG in between pH 6–13 and in between pH 3–13 for PMEE-PDTriG. Higher solubility of the reported polymers was also achieved by influence of temperature, making them polymeric-thermometers as they are thermosensitive in aqueous media. Photophysical properties at different pH values in a broad temperature window (10–80 °C) shows that there is a temperature response for both polymers. Moreover, stability of the polymers was maintained throughout multiple heating–cooling cycles. Furthermore, UCST-like thermoresponsive behavior was demonstrated for PDTriG with Ca^2+^ ions, which are known to bind divalently with carboxylate groups. The combined pH-responsive and thermoresponsive properties of these PPVs are promising features for the design of sensors, specifically for their use as dual sensors for temperature and pH values. Such dual sensors are a highly desired addition to the class of polymeric sensors and a related system based on a temperature-responsive polymer with a pH-responsive solvatochromic dye in the side chain. The hysteresis gap in the heating–cooling cycles at lower temperatures for PMEE-PDTriG brings about an optional application of a solution thermometer with memory function.

## Figures and Tables

**Figure 1:**
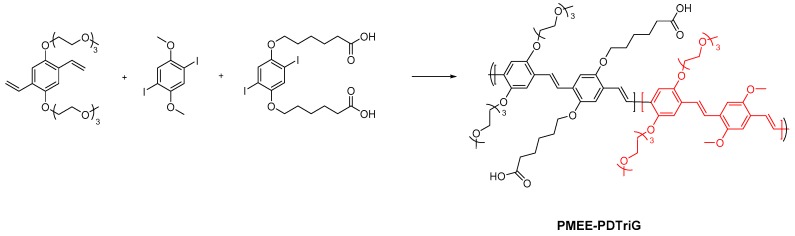
Synthetic scheme of PMEE-PDTriG.
